# Disrupting EGFR–HER2 Transactivation by Pertuzumab in HER2-Positive Cancer: Quantitative Analysis Reveals EGFR Signal Input as Potential Predictor of Therapeutic Outcome

**DOI:** 10.3390/ijms25115978

**Published:** 2024-05-29

**Authors:** László Ujlaky-Nagy, János Szöllősi, György Vereb

**Affiliations:** 1 Department of Biophysics and Cell Biology, Faculty of Medicine, University of Debrecen, Egyetem tér 1, H-4032 Debrecen, Hungary; 2 HUN-REN-UD Cell Biology and Signaling Research Group, Faculty of Medicine, University of Debrecen, Egyetem tér 1, H-4032 Debrecen, Hungary; 3 Faculty of Pharmacy, University of Debrecen, Egyetem tér 1, H-4032 Debrecen, Hungary

**Keywords:** epidermal growth factor receptor, EGFR, HER2, ErbB2, fluorescence correlation spectroscopy, FCS, fluorescence cross-correlation spectroscopy (FCCS), confocal microscopy, Förster resonance energy transfer (FRET), pertuzumab (Perjeta^®^), trastuzumab (Herceptin^®^, Trazimera^TM^), targeted antibody therapy

## Abstract

Pertuzumab (Perjeta^®^), a humanized antibody binding to the dimerization arm of HER2 (Human epidermal growth factor receptor-2), has failed as a monotherapy agent in HER2 overexpressing malignancies. Since the molecular interaction of HER2 with ligand-bound EGFR (epidermal growth factor receptor) has been implied in mitogenic signaling and malignant proliferation, we hypothesized that this interaction, rather than HER2 expression and oligomerization alone, could be a potential molecular target and predictor of the efficacy of pertuzumab treatment. Therefore, we investigated static and dynamic interactions between HER2 and EGFR molecules upon EGF stimulus in the presence and absence of pertuzumab in HER2+ EGFR+ SK-BR-3 breast tumor cells using Förster resonance energy transfer (FRET) microscopy and fluorescence correlation and cross-correlation spectroscopy (FCS/FCCS). The consequential activation of signaling and changes in cell proliferation were measured by Western blotting and MTT assay. The autocorrelation functions of HER2 diffusion were best fitted by a three-component model corrected for triplet formation, and among these components the slowly diffusing membrane component revealed aggregation induced by EGFR ligand binding, as evidenced by photon-counting histograms and co-diffusing fractions. This aggregation has efficiently been prevented by pertuzumab treatment, which also inhibited the post-stimulus interaction of EGFR and HER2, as monitored by changes in FRET efficiency. Overall, the data demonstrated that pertuzumab, by hindering post-stimulus interaction between EGFR and HER2, inhibits EGFR-evoked HER2 aggregation and phosphorylation and leads to a dose-dependent decrease in cell proliferation, particularly when higher amounts of EGF are present. Consequently, we propose that EGFR expression on HER2-positive tumors could be taken into consideration as a potential biomarker when predicting the outcome of pertuzumab treatment.

## 1. Introduction

The HER transmembrane receptor tyrosine kinase family (human epidermal growth factor receptor, also known as ErbB) has four members, HER1 (EGFR) through HER4. The family members can form different homodimers, heterodimers, and oligomers induced by specific ligands, which recruit various downstream adapters, resulting in the activation of diverse signal transduction pathways. These play substantial roles during embryo- and organogenesis by determining the development and fate of cells [1].

The most preferred hetero-dimerization partner of the family members is HER2 due to its constitutively active conformation [2], which precludes the need for an activating ligand. In coherence with this, HER2 does not actually have a known ligand [3]. This renders HER2 a ubiquitous shared co-receptor, which broadens ligand-responsiveness by hetero-dimerization and provides the most potent mitogenic signals [4,5]. Mutations of HER2 or overexpression of signaling components of the HER2 pathway have been found in several malignancies [6]. HER2 gene amplification in breast cancer is closely related to tumor-cell proliferation and invasion, resulting in focal progression and distant metastases [7,8]. Aberrations in HER2 signaling were found in 20% of breast cancer [9], highlighting the fact that HER2 status plays important role as a prognostic factor (reviewed in [10]).

All HER proteins, except for EGFR, show impaired ligand-dependent down-regulation and degradation by endocytosis [11], which may affect their signaling potency [12]. HER2 prevents EGFR from endocytotic degradation causing increased recycling [11]. At the same time, HER2 overexpression results in enhanced heterodimerization with EGFR [13,14], leading to increased signaling activity [12,15,16,17].

Several breast cancers overexpress HER2, providing a potential target for immunotherapy [18,19]. The first humanized monoclonal antibody applied in solid tumors, trastuzumab, targets HER2 [20]. Its ability to inhibit in vitro and in vivo tumor growth [21] is attributed to internalization and down-regulation of cell surface HER2 [22], inhibition of the PI3K/Akt pathway [23], cell cycle arrest in G1, inhibition of angiogenesis [24], and antibody-dependent cell-meditated cytotoxicity (ADCC) [25,26,27].

Based on the success of trastuzumab, another anti-HER2 monoclonal antibody, pertuzumab, has also been developed for treating patients with HER2-positive breast cancer [28]. It is a recombinant humanized IgG1 binding extracellularly to HER2 subdomain II, resulting in the inhibition of ligand-induced HER2 dimerization [29,30,31,32,33]. Although this proposed mode of action has not yielded clinical success in trials of pertuzumab as monotherapy (trial NCT02491892), it was later shown that adding pertuzumab to the then standard-of-care trastuzumab plus a chemotherapy regime improves clinical outcome [34]. Consequently, the FDA granted regular approval to pertuzumab in combination with trastuzumab and chemotherapy for the adjuvant treatment of patients with HER2-positive early breast cancer at high risk of recurrence [35]. However, the beneficial effects [36,37] of the combination appear to be based on the additive effect of the two antibodies on enhancing ADCC [36,37], and thus the molecular profiles and molecular interactions in tumors that could represent a target for the direct action pertuzumab remain speculative.

Such interactions are routinely revealed in bulk in vitro samples by immunoprecipitation; however, to show that they occur in situ in the cell and could possibly be disrupted by a pharmacological agent requires sensitive microscopic approaches [38]. Fluorescence correlation spectroscopy (FCS) and fluorescence cross-correlation spectroscopy (FCCS) are advanced methods with potential single molecule sensitivity that have currently started to gain popularity due to their potential for sensitively assessing molecular mobility and dynamic interactions in situ in living cells [39,40,41,42,43,44]. Since alterations in molecular mobility and consequential changes in characteristic diffusion times are not highly sensitive to dimerization or low levels of aggregation, these associations are best monitored through the photon-count histogram (PCH), which is highly sensitive to oligomerization [45,46]. Additionally, the proportion of co-diffusing species assessed by FCCS can also be an effective descriptor of stable molecular interactions.

We have used these approaches, as well as Förster resonance energy transfer (FRET) [47,48,49], to explore how EGF binding to its receptor transactivates HER2, and whether there is direct evidence for pertuzumab inhibiting this transactivation. Our model system was the SK-BR-3 cell line, having a moderate level of EGFR and a higher level HER2 expression. Data show that pertuzumab hinders post-stimulus interaction between EGFR and HER2, inhibits EGFR-evoked HER2 aggregation and phosphorylation, and leads to a dose-dependent decrease in proliferation. Consequently, we propose that EGFR expression on HER2-positive tumors could be used as a biomarker for identifying tumors that are potentially sensitive to the direct biological effects of pertuzumab.

## 2. Results

### 2.1. Molecular Interaction of EGFR and HER2 and Its Modulation by EGF and Pertuzumab

**Figure 1:**
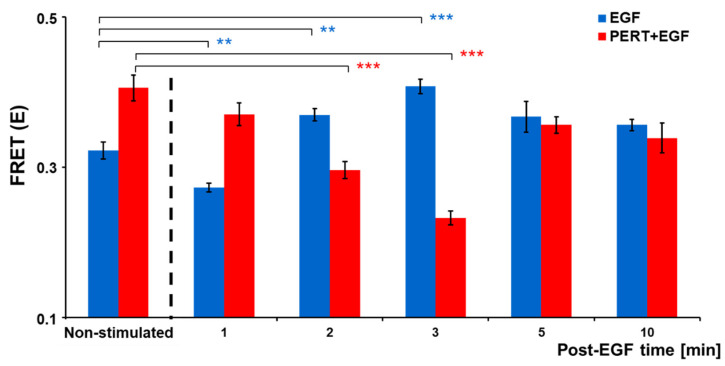
Changes in the interaction between EGFR and HER2 induced by EGF in the absence and presence of pertuzumab. From microscopic images of fluorescently labeled SK-BR-3 cells, FRET efficiency was measured between EGFR (donor) and HER2 (acceptor) using intensity based (ratiometric imaging) FRET before and 1, 2, 3, 5, and 10 min after stimulation with 100 nM EGF. Samples were pre-treated with 50 μg/mL (final concentration) of pertuzumab for 20 min. FRET efficiency values, calculated from >58 cells per treatment (2 independent experiments, total 1163 cells) are mean ± SEM. After two-way ANOVA revealed significant interaction between factors, one way ANOVA with Dunnett’s post hoc test was used for statistical comparison (** *p* < 0.01; *** *p* < 0.001).

Since EGF-bound EGFR is a preferred heterodimerization partner of HER2, we applied FRET to map their molecular interaction. Samples were fixed at various time points after EGF stimulus and fluorescently labeled, and FRET efficiency was determined using the spectrally corrected, 3-filter-based ratiometric imaging approach (RiFRET) in a confocal microscope [49]. To assess its inhibitory potential on ligand-induced di-/oligomerization, pertuzumab was applied as a pretreatment. The baseline FRET efficiency revealed that a significant proportion of EGFR is in molecular proximity of HER2 in resting cells, which transiently decreased and then, from 2 min post-stimulus, significantly increased after adding EGF, reaching its peak at 3 min. Pertuzumab pretreated cells showed higher EGFR–HER2 FRET at baseline, which decreased upon EGF stimulus, reaching its minimum at 3 min post-stimulus, and then increased by 5 and 10 min. Notably, at 2 and 3 min after the addition of EGF to serum-starved cells, the presence of pertuzumab significantly decreased EGFR–HER2 interaction (Figure 1).

### 2.2. Membrane Dynamics of HER2 Revealed by FCS

**Figure 2:**
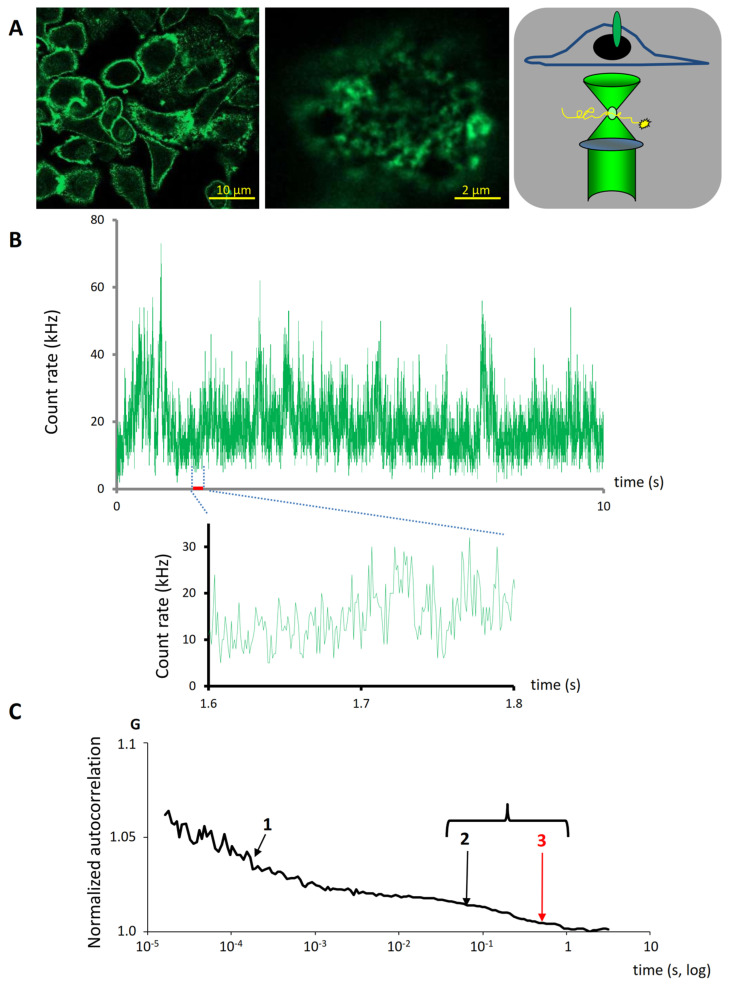
Fluorescence correlation spectroscopy revealed different membrane components of HER2 molecules based on their diffusion characteristics. Membrane HER2 was labeled with Fab fragments of the anti-HER2 monoclonal antibody 76.5, conjugated with Alexa-Fluor 488. (**A**) Confocal images were taken from labeled SK-BR-3 cells. After a z-scan, a membrane section distal from the chamber bottom was selected and the confocal excitation volume of FCS was set to overlap with it (far right scheme). (**B**) The fluctuation in fluorescence intensity produced by the diffusion of labeled HER2 molecules in and out of this sub-femtoliter volume was measured and analyzed. The curves show typical time traces with low and high temporal resolution. Background intensity was assessed to be ~2 kHz with 488 nm excitation. (**C**) The generated autocorrelation curves were fit with a tree-component diffusion model. The fastest component (1) was attributed to unbound Fab, and the other two components (2, 3) to labeled membrane HER2 molecules.

The diffusion characteristics of HER2 molecules in SK-BR-3 cells were monitored using fluorophore conjugated Fab-s bound to the extracellular domain of HER2. FCS spectra were obtained at a Z-position where the observation volume overlapped the most with the cell membrane distal from the substrate, as evidenced by the highest fluorescence intensity (Figure 2A, far right scheme). From these spectra, autocorrelation curves were generated. The best fit to these curves revealed a three-component diffusion model also incorporating triplet state (Figure 2). In this model, two two-dimensional (2D) membrane diffusion components were present; the faster 2D component was attributed to monomers or smaller oligomers, while the slower 2D component was attributed to larger aggregates. Additionally, a very fast 3D diffusion component was attributed to free Fab fragments. Importantly, by fitting the autocorrelation curves, the number of diffusing species in the observation confocal volume was determined, and from this their concentration can also be determined. The fit function was consecutively used to quantitatively analyze aggregation and consequential stable co-diffusion of HER2 molecules under various treatment conditions.

### 2.3. Fluorescence Cross-Correlation Spectroscopy Sensitively Detects the Fraction of Stably Co-Diffusing HER2 Molecules

**Figure 3:**
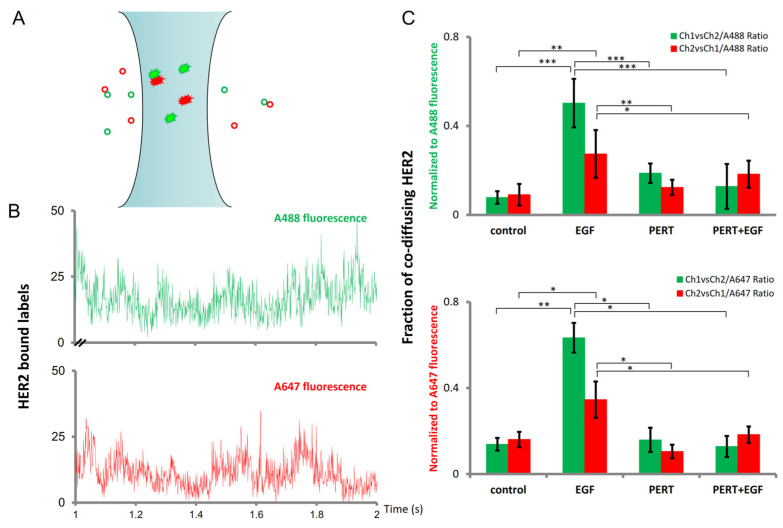
Fluorescence cross-correlation spectroscopy (FCCS) reveals transactivation-induced HER2 oligomerization and its inhibition by pertuzumab. Membrane HER2 molecules were labeled simultaneously with a mixture of Fab fragments, conjugated either with Alexa-Fluor 488 or with Alexa-Fluor 647. (**A**). Certain HER2 molecules carrying spectrally distinct labels may enter and exit the observation volume together, while others diffuse independently (**B**). Sample time traces of the two distinct spectral channels representing partially synchronous fluctuations in time originating from the stable co-diffusing species. (**C**) The column charts represent the fractional distribution of slowly co-diffusing HER2 (±SD) normalized to the total amount of receptors in either one of the individual channels (Alexa-Fluor 488 or Alexa-Fluor 647), before (*n* = 14) (control) and after (*n* = 9) 100 nM EGF (EGF), as well as after pertuzumab pretreatment (*n* = 30) (PERT) and following EGF stimulus after pertuzumab pretreatment (*n* = 8) (PERT + EGF). Correlation spectra were collected starting 5 min post stimulus. ANOVA with Dunnett’s post hoc test was used to compare treatment conditions (significance levels: *p* < 0.05 (*); *p* < 0.01 (**); *p* < 0.001 (***)).

Simultaneous excitation and recording of fluorescence intensity fluctuations derived from spectrally different fluorescent species allows not only the calculation and fitting of the autocorrelation function for each labeled species, as described previously, but also, in the same measurement, a fluorescence cross-correlation function (Figure 3). These latter yield information on the concentration of the stably co-diffusing entities, while the concentration of each labeled species is derived from their autocorrelation functions. Using these data, the relative fraction of each species co-diffusing with the other can be calculated. By labeling target HER2 molecules with a mixture of Fab-s conjugated either with Alexa-Fluor 488 or Alexa-Fluor 647 dyes, the aggregation state of HER2 can be judged from the relative co-diffusing fraction. Because the cross-correlation function can be calculated with either of the fluorescence channels being the reference, the obtained values can be normalized to either of the autocorrelation channels; in Figure 3C, all the possible four co-diffusing fractions are presented.

These data unanimously imply that EGF-induced transactivation increased the fraction of co-diffusing HER2. Pertuzumab pretreatment for 20 min alone did not significantly change this fraction as compared to control; however, it almost completely abolished the EGF-induced increase in co-diffusion.

### 2.4. Photon-Count Histogram (PCH) Analysis of HER2 Oligomerization

**Figure 4:**
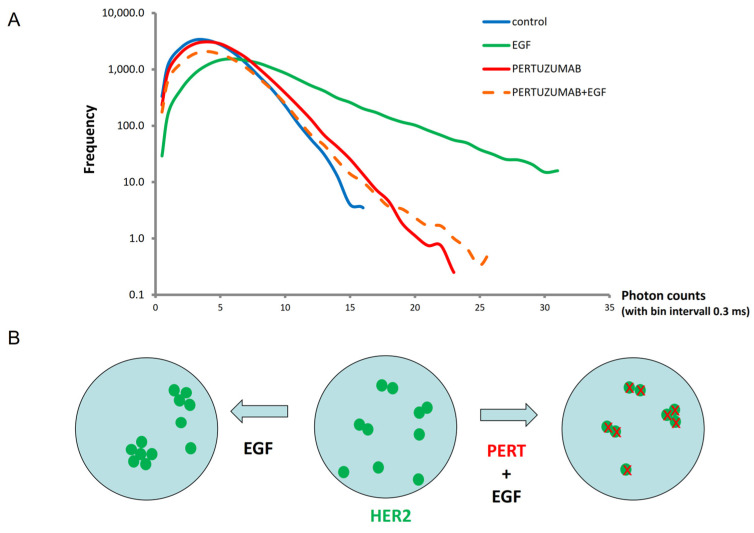
PCH analysis confirms higher-order aggregation of EGFR-transactivated HER2 and demonstrates the blocking of this aggregation by pertuzumab in situ in cells. (**A**) Frequency distribution of average photon count (bin interval 300 μs). Higher-order aggregates result in higher photon counts per diffusing particles, leading to broadening and shifting to the right of the PCH curve. Samples plotted: control (blue), stimulated with EGF (5 min, 100 nM, green), pre-incubated with pertuzumab (20 min, 50 μg/mL, red), and treated with EGF after pertuzumab pre-incubation (dashed orange). (**B**) Schematic diagram of EGF increasing the size of HER2 aggregates, and the blocking (x) of this increase by pertuzumab pretreatment.

Although FCCS proved to be sufficiently sensitive for demonstrating key changes in HER2 oligomerization, it is only a proxy to estimating the degree of aggregation, since it cannot reveal whether several smaller oligomers, or fewer larger aggregates, co-diffuse. Photon-counting histogram (PCH) analysis and representation could help to resolve the heterogeneity of aggregation states more effectively at the single-molecule level through assessing species brightness [46,50,51,52]. To exploit PCH for characterizing the extent of aggregation more precisely, we have used Fab fragments conjugated with Alexa-Fluor 488 for labeling HER2 and measured the fluctuation of fluorescence in the membrane. From the fluctuation traces, we have constructed photon-count histograms (PCH, Figure 4). These histograms show the frequency distribution of photon counts, which characterizes the average brightness of diffusing particles over a short time window. The control cells showed a distribution histogram having a mode of 3 and a maximum of 16 photons per particle. EGF treatment has caused a high degree of aggregation, resulting in a mode of 6 and a maximum of 32, implying that the size of the most frequent aggregate has doubled, and the largest aggregates were also twice as large. Pretreatment with pertuzumab only slightly increased the mode and the maximum measured for the control, and these values essentially did not further change upon adding EGF. Taken together with the FCCS data, the addition of EGF increases not only the fraction of co-diffusing HER2 but also the size of its aggregates, and this is completely prevented by pertuzumab pretreatment (Figure 4B).

### 2.5. Western Blot Analysis of HER2 Transactivation by EGF Bound EGFR

**Figure 5:**
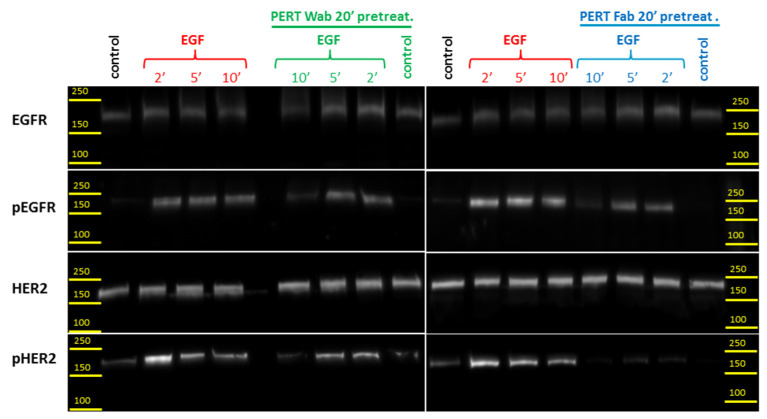
Functional consequences of EGFR–HER2 interaction manifested in receptor phosphorylation. EGFR and HER2 phosphorylation are shown on Western blot. Samples were stimulated with 100 nM EGF for 2, 5, and 10 min. Pre-incubation with pertuzumab, or pertuzumab Fab (20 min, 50 μg/mL) was also applied and compared to controls.

To characterize the immediate consequences on HER2 signaling of EGF-triggered transactivation and the potential modulatory effects of pertuzumab, EGFR, HER2, and their activated phosphorylated forms were detected in Western blots. Moderate baseline phosphorylation of HER2 was found in control samples (Figure 5). When an EGF stimulus was applied, the amount of activated, phosphorylated EGFR increased rapidly. In parallel, due to HER2 transactivation, the amount of phosphorylated HER2 also increased. As post-stimulus time progressed, dephosphorylation of the receptors ensued by 10 min.

Transactivation of HER2 was inhibited by pre-incubation with pertuzumab (whole antibody) and to an even greater extent with its Fab fragment. The co-temporal decrease in EGFR phosphorylation well demonstrates that EGFR–HER2 transactivation is not a unidirectional process; activated HER2 also activates EGFR, and this is also diminished by pertuzumab.

### 2.6. Pertuzumab Inhibits the Growth of EGFR and HER2 Double-Positive Cells In Vitro

**Figure 6:**
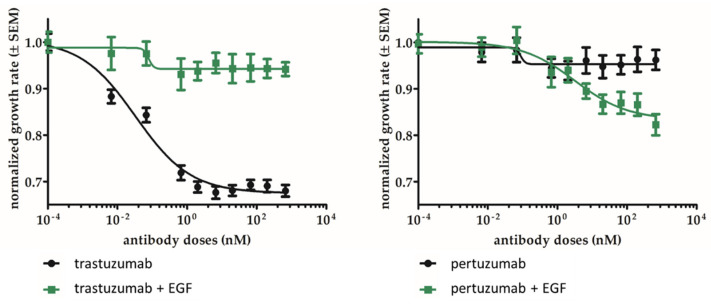
Sensitivity of EGFR and HER2 double-positive SK-BR-3 to pertuzumab and trastuzumab with or without supplementary EGF. An MTT assay was applied to show long-term effects on the total metabolic activity of cell the cell culture. Supplementary EGF-stimulus was 100 nM. Growth inhibition was plotted as a function of applied antibody concentration (nM) and fitted to the Hill equation. Data are normalized to untreated cells for antibody treatment and to EGF treated cells for EGF + antibody treatment.

The SK-BR-3 cell line we used expresses a moderate level of EGFR and overexpresses HER2. The inhibitory effect of pertuzumab on its proliferation was assessed through bulk metabolic activity using an MTT-based assay. For reference, we have also used trastuzumab treatment, which is known to inhibit the proliferation of these cells. Both trastuzumab and pertuzumab exerted a dose-dependent antiproliferative effect, and as previously observed [36,37], SK-BR-3 was more sensitive to trastuzumab in terms of overall growth reduction (Figure 6, black traces). However, IC50 values (trastuzumab 0.033 nM, pertuzumab 0.087 nM) were not statistically different. Importantly, excess EGF supplied to the culture medium in addition to the EGF content of the fetal calf serum increased proliferation rate by ~55%, and this increased; EGF-dependent proliferation was more efficiently inhibited by pertuzumab then by trastuzumab (Figure 6, green traces). 

## 3. Discussion

Amplification of HER2 occurs in 20–25% of breast cancers and is associated with an aggressive tumor phenotype, elevated risk of recurrence, and poor prognosis [10]. Since hetero-dimerization of HER2 with ligand-bound EGFR is an important signal input for cells expressing both receptors [1], which may have implications on humanized antibody therapies specifically targeting the dimerization arm of HER2 [29,30,31,32,33], we have set out to quantitatively analyze EGF-driven HER2 transactivation at the levels of molecular dynamics in the cell membrane, consecutive phosphorylation, and cell proliferation, and to examine how these are influenced by pertuzumab, the first humanized anti-HER2 monoclonal antibody that inhibits HER2 dimerization.

First, we characterized the molecular interaction of EGFR and HER2 in the membrane of SK-BR-3 mammary carcinoma cells that express these receptors at low and high levels (~80,000 and 800,000 per cell, respectively). We have used the spectral overlap-corrected three-filter-based Förster resonance energy transfer (FRET) method in confocal microscopy to assess the level of heterodimerization. This approach has revealed spontaneous hetero-aggregation between EGFR and HER2 on resting, serum starved cells. As expected in this model, EGF treatment of serum starved cells increases EGFR–HER2 interaction significantly after a transient decrease at 1 min; this latter is likely attributable to the initial increase in EGFR homodimerization at the expense of heterodimerization upon EGF-ligand binding. A higher baseline FRET in pertuzumab pretreated cells may represent the joint effect of pertuzumab crosslinking HER2 together with an excessive cell surface presence of EGFR owed to serum withdrawal. Importantly, there is a prominent, significant decrease in EGFR–HER2 interaction in pertuzumab-pretreated cells at 2 and 3 min post-stimulus, which coincides with the expected peak of transactivation, both signified by the highest FRET efficiency values in EGF-stimulated cells (Figure 1) and the corresponding highest EGFR and HER2 phosphorylation (Figure 5). Beyond 10 min post stimulus, apparently a new equilibrium is being established, with heterodimerization of control cells decreasing, but this is less influenced by pertuzumab, which is not replenished after receptor internalization and recycling.

Since FRET is only capable of providing a snapshot of molecular associations, we next used fluorescence correlation/cross-correlation spectroscopy (FCS/FCCS) to characterize stable associations that manifest in the co-diffusion of molecules during the observation time. Analysis of autocorrelation curves revealed different subsets of HER2 molecules based on their diffusion. These subpopulations co-exist in the membrane of unstimulated cells. Fast-membrane components represent mainly HER2 monomers/dimers, and possibly some smaller oligomers, based on their characteristic diffusion time, similar to the transferrin receptor and other monomeric membrane proteins [53]. The slow membrane component can be attributed to the diffusion of various larger HER2 oligomers or clusters that are still small enough to move through the observation volume within the acquisition time (in the range of seconds). To be able to resolve these two membrane components, a much faster diffusion correlation time (<10 ms) in 3D had to be assumed and fitted, which corresponds to the free diffusion of unbound Fab fragments, as evident from measuring the cell-free labeling buffer. 

Having optimized the fitting model, we measured the co-diffusing fraction of the oligomeric membrane component using FCCS. This approach proved to be sufficiently sensitive in revealing that a large, 30–65% fraction of HER2 molecules stably co-diffuses on the several hundred ms timescale during the first few minutes after EGF stimulus, as opposed to resting cells where this fraction is 10–20%. Pretreatment with pertuzumab alone caused no significant shift from control, suggesting that stable co-diffusion of HER2 molecules is not altered by pertuzumab, even though single-cell flow cytometric FRET measurements have revealed that the average distance of labeled HER2 molecules is decreased in these cells [54]. Importantly, however, pertuzumab pretreatment nearly completely abolished the EGF-evoked increase in the co-diffusing fraction.

As the co-diffusing fraction does not directly translate into the level of receptor aggregation, we have also exploited photon-count histogram analysis to assess the brightness distribution of diffusing species, which, assuming that binding of the Fab label is proportional to the number of HER2 molecules in a cluster, is a good indicator of oligomerization. PCH histograms evidenced a doubling of HER2 cluster size upon EGF stimulus over the first 5 min, which was inhibited by pertuzumab pretreatment, coherent with our observations on the co-diffusing fraction.

Next, we explored, using Western blotting, how EGF-induced HER2 aggregation translates into EGFR and HER2 activation as reflected by the phosphorylation of these receptors. EGF stimulation caused an increase in both EGFR and HER2 phosphorylation, which then decayed close to baseline in 10 min. The extreme fast kinetics of HER2 phosphorylation reaching maximum at approximately 2 min, and then switching over to dephosphorylation, hint that a small number of ligand-bound EGFRs may act as a signal-antenna, rapidly forwarding the signal to HER2, a significant proportion of which is in preformed HER2 di- and oligomers on the cell surface and which then undergoes further aggregation, as evidenced by PCH analysis. Not surprisingly, pertuzumab pretreatment potently inhibits the transactivation and consequential HER2 autoactivation process. Of note, Fab fragments of pertuzumab were more efficient in decreasing transactivation signaling than whole antibodies. We assume that its smaller size makes its binding to the dimerization arm of HER2 easier. An interesting additional finding is that pertuzumab also inhibits EGFR phosphorylation, although it does not bind to EGFR, which underlines the importance of activated HER2 reciprocally activating EGFR, which further contributes to the rapid kinetics of activation and phosphorylation.

Finally, we have confirmed that pertuzumab inhibits the proliferation of HER2/EGFR double-positive breast cancer SK-BR-3 cells similarly to the clinically successful trastuzumab. The extent of inhibition was moderate, which is coherent with earlier findings [55]. While the IC50 concentrations are not significantly different for the two antibodies, in the case of cells grown in regular medium the overall decrease in proliferation rate is significantly smaller for pertuzumab (~5%) than for trastuzumab (~30%). However, when the medium is supplemented with extra EGF, mimicking a high local ligand concentration in tumors with auto/paracrine signaling, pertuzumab becomes more efficacious, reaching ~20% inhibition. This highlights the possibility that EGFR expression on HER2-positive tumors could be used as a potential biomarker for predicting the outcome of pertuzumab treatment. 

The limitation of this study is the simplified cellular system, which allows focusing on the transactivation of HER2 by EGFR without having to consider additional crosstalk from the tumor microenvironment (TME), circulating growth factors, and the diverse functions of the immune system. 

An important aspect of the TME is that it is a source of growth arrest-specific 6 (GAS6), which is the ligand of the AXL kinase and can provide another distinct transactivation pathway for HER family kinases expressed on tumor cells [56]. GAS6 upregulation or AXL overexpression is often found in cancers with resistance to EGFR-targeted therapy and enhanced survival [57]. Activation of AXL requires phosphatidyl serine for GAS6 following its posttranslational modification [58], and the tumor microenvironment contains a high level of externalized phosphatidyl serine due to the increased apoptotic index of tumors, metabolically stressed tumor cells, vasculature within the tumor, and tumor-derived exosomes [56,59]. In HER2+ breast cancers, AXL can interact with, and is phosphorylated by, HER2, especially when HER2 is overexpressed [60]. Although AXL is present in SK-BR-3 cells, owing to the lack of GAS6 in our in vitro system this interaction is not expected to interfere with EGFR–HER2 transactivation and its inhibition by pertuzumab.

Interestingly, the signal from AXL may induce low-fidelity DNA polymerases and Myc, thereby evolving tolerance towards endogenous mutators and antibiotics, as well as drugs in cancers. Blocking or the ablation of AXL has the potential to restore drug-sensitivity, which provides new combination strategies against EGFR/HER-expressing tumors [61]. The development of AXL-targeted inhibitors is dynamic [62,63,64,65,66]. Although there are promising clinical trials and studies targeting AXL [67,68], redundancy of signaling through other TAM (TYRO3, AXL, and MERTK) family kinases may decrease their effectiveness [69]. These phenomena invoke the combination of anti-EGFR/HER treatments with TAM inhibitors to replace monotherapy [70,71,72,73,74,75] as well as the development of pan-TAM inhibitors [76,77]. Clinical resistance to AXL-targeting therapies may also involve HER3 [78], but this requires further investigation [79,80]. 

However, HER3 has been long implicated in HER2 transactivation, and as such has received much attention, both mechanistically [81] and in terms of therapeutic relevance [82]. Although in HER2+ breast cancer cell lines the expression and constitutive phosphorylation of HER3 are frequent, a high variability was observed in this respect in the HER2-amplified cancers of other tissues, and the level of HER3 expression was not a good predictor of its functional significance [83]. This is in line with the finding that, out of a cohort of 29 HER3+ clinical tumors, only one was responsive to the novel HER3-targeting monoclonal antibody GSK2849330, and this was the case of a CD74-NRG1-rearranged lung cancer, underlining the importance of signaling input through HER3 by this neuregulin fusion protein [84]. This and similar findings have led to the currently running phase I/II trial in various NRG1 fusion-positive cancers of Zenocutuzumab, a novel, bispecific antibody binding both HER2 and HER3 and inhibiting NRG1 binding through a ‘Dock & Block^®^’ mechanism of action [85]. In our model, even though SK-BR-3 expresses HER3, the absence of a specific ligand helps avoid the confounding effect of HER3–HER2 transactivation. Such an effect could only be achieved in transgenic SK-BR-3 expressing heregulin [86]. Even in these cells, the combination of trastuzumab and pertuzumab had no deleterious effect on cell proliferation, and only their further combination with the anti-HER3 antibody patritumab was effective [86]. Very recently, the antibody–drug conjugate (ADC) of this antibody with deruxtecan, HER3–DXd, was found safe and effective in heavily pretreated patients with diverse HER3-positive tumors; however, the effect was independent of the presence of HER2 on these tumors [87]. 

It is important to note, however, that the success of targeted antibody treatment depends upon multiple mechanisms of action, and in addition to direct biological effects on the tumors, the recruitment of antibody-dependent immune mechanisms such as ADCC are also a major mode of their action [36,37,88], and clinical success is best predicted by comprehensive analysis of all the different impacts, including the capacity for exploiting the bound therapeutic antibody in ADCC [89]. 

## 4. Materials and Methods

All materials were from Sigma-Aldrich, St. Louis, MO, USA, unless otherwise indicated.

### 4.1. Cell Culture

The SK-BR-3 cell line was obtained from the American Type Culture Collection (ATCC, Rockville, MD, USA) and cultured according to specifications. Cells were cultured in a humidified atmosphere with 5% CO_2_ at 37 °C in Dulbecco’s Minimal Essential Medium (DMEM) supplemented with 10% fetal calf serum (FCS) and with gentamycin as antibiotic. The SK-BR-3 cells express a relatively small amount of EGFR (80,000 molecules per cell) but overexpress HER2 molecules (800,000 per cell), as confirmed using a Qifikit (Dako, Glostrup, Denmark) standard. 

Cells were propagated every 2–3 days. Cells were seeded either onto coverslips, into 8-well ibidi chambers, 96-well tissue culture plates, or petri dishes 36–48 h before use. Prior to stimulation in all types of experiments (except metabolic activity assay), cells were kept for 2 h in serum-free HEPES buffer supplemented with 10 mM glucose. For MTT-based metabolic assays, indicator-free medium was used.

### 4.2. Concofal Microscopy Imaging and Spectrally Corrected Three-Filter (Ratiometric) FRET Imaging (riFRET)

Cells, seeded previously onto coverslips, were starved in serum-free HEPES buffer (20 mM HEPES, 123 mM NaCl, 5mM KCl, 1.5 mM MgCl2, 1 mM CaCl2, 10 mM glucose, pH 7.2) for 2 h before samples were stimulated with EGF (at a saturation dose of 100 nM) for 2 and 5 min. Pertuzumab (Perjeta^®^, Roche Holding AG, Basel, Switzerland) pretreatment was performed in HEPES buffer at 37 °C for 20 min before labeling or stimulating with EGF. Cells were labeled with anti-EGFR ab-11 mouse monoclonal antibody (20 μg/mL) (Thermo Fisher Scientific, Waltham, MA, USA) on ice for 15 min. The non-competing nature of this Mab with EGF binding was verified using A431 cells that overexpress EGFR at ~1 million copies (Figure S1). After washing with ice-cold HEPES buffer, cells were indirectly labeled with Alexa-Fluor 546 conjugated goat-α-mouse secondary antibody Fab (20 μg/mL) for 15 min at 0 °C). FMO controls by omitting the primary antibody showed no fluorescence above background. In parallel with the 2nd labeling step, cell-surface HER2 molecules were labeled with Alexa-Fluor 647 conjugated trastuzumab (20 μg/mL final concentration) (Herceptin, Roche Holding AG, Basel, Switzerland), on ice for 15 min. Then, cells were washed and fixed with formaldehyde (2%) for 20 min (10 min fixation at 4 °C, then warming gradually to room temperature). Samples were embedded into antifade media (Mowiol) and were examined with a Zeiss LSM 880 using a C-Apochromat 40×/1.2 W objective (Carl Zeiss AG, Oberkochen, Germany). Scanning parameters and filters were set according to the applied fluorophores: 543 nm exc/548–631 nm em ($I_{1}$, donor signal), or 642–695 nm em ($I_{2}$, FRET signal); 633 nm exc/642–695 nm em ($I_{3}$, acceptor signal). Pixel size and sampling was set to Nyquist criteria. A diffraction-limited optical slice of the ‘upper’ cell membrane, distal from the culture dish surface, was taken with the pinhole set to 1 Airy unit. Further image analysis was performed in Zeiss Zen Black 2.1 (Carl Zeiss AG, Oberkochen, Germany) and Fiji/ImageJ 1.54i. The average FRET efficiency for each cell’s top membrane area was calculated from pixels with donor intensity above background and used for statistical calculations.

Pixel-by-pixel FRET efficiency was evaluated according to the following equations using the RiFRET plugin we formerly developed [49,90]:

$$E=\frac{S_{2}\left(I_{2}-I_{1}S_{1}-I_{3}S_{2}+I_{1}S_{2}S_{3}+I_{3}S_{1}S_{4}-I_{2}S_{3}S_{4}\right)}{\alpha \left(\frac{\varepsilon_{\lambda D}^{A}\varepsilon_{\lambda A}^{D}}{\varepsilon_{\lambda A}^{A}\varepsilon_{\lambda D}^{D}}-1\right)\left(I_{2}S_{4}-I_{1}S_{2}\right)+S_{2}\left(I_{2}-I_{1}S_{1}-I_{3}S_{2}+I_{1}S_{2}S_{3}+I_{3}S_{1}S_{4}-I_{2}S_{3}S_{4}\right)}$$

$I_{1}$; $I_{2}$; $I_{3}$: fluorescence intensity of donor (1, exc_donor_-em_donor_) transfer (2, exc_donor_-em_acceptor_) and acceptor (3, exc_acceptor_-em_acceptor_) channels from dual-labeled samples; $\varepsilon_{\lambda D}^{A}$: molar extinction coefficient of acceptor at the donor excitation wavelength; $\varepsilon_{\lambda A}^{D}$: molar extinction coefficient of donor at the acceptor excitation wavelength; $S_{1}$; $S_{3}$; spillover factors determined from donor-only labeled samples and calculated as $S_{1}=\frac{I_{2}^{Donor}}{I_{1}^{Donor}}$, $S_{3}=\frac{I_{3}^{Donor}}{I_{1}^{Donor}}$; $S_{2}$,$S_{4}$ spillover factors determined from acceptor-only labeled samples and calculated as $S_{2}=\frac{I_{2}^{Acc}}{I_{3}^{Acc}}$, $S_{4}=\frac{I_{1}^{Acc}}{I_{3}^{Acc}}$.

α: correction factor for detection efficiency, calculated as:

$$\alpha =\frac{I_{2}^{Acc}\varepsilon_{\lambda D}^{D}L_{D}B_{D}}{I_{1}^{Donor}\varepsilon_{\lambda D}^{A}L_{A}B_{A}}$$

$I_{2}^{Acc}$: fluorescence intensity of acceptor-only sample in transfer channel; $I_{1}^{Donor}$: fluorescence intensity of donor-only sample in the donor channel; $L_{i}$: labeling ratio of the applied antibodies; $B_{i}$: mean number of receptors per cells labeled by the donor or acceptor antibody, respectively.

### 4.3. Fluorescence Correlation and Cross-Correlation Spectroscopy

After setting the excitation volume on the upper cell membrane, the diffusion characteristics of fluorescently labeled HER2 molecules were assessed in live SK-BR-3 cells previously seeded into 8-well chambers (Ibidi, 0.17 mm bottom thickness) at a density of 40,000/cm^2^ (sub-confluence) and grown for 24–48 h to a confluence level of 70% to 90%. Cells were starved in serum-free HEPES buffer for 2 h before the experiment and were labeled with Alexa-Fluor 488 or Alexa-Fluor 647 conjugated mouse 76.5 Fab fragments (1 µg/mL—experimentally titrated for optimal brightness) for 5 min. (The hybridoma producing 76.5 was a kind gift from Yosef Yarden, Weizmann Inst. of Science, Rehovot, Israel.) Antibody purification and Fab fragment preparation were performed in our laboratory according to standard protocols. Antibody binding and specificity was confirmed in comparison to trastuzumab and pertuzumab (Figure S2). Unbound excess Fab molecules were removed by washing the cells twice with HEPES buffer at 37 °C. The applied 76.5 Fab fragment did not induce internalization in the time range of the experiments. Prior to FCS experiments, confocal images were taken of the fluorescently labeled SK-BR-3 cells to determine the position of membrane sections distal from the chamber’s bottom. A Z-line scan was then acquired to set the subfemtoliter confocal excitation volume to overlap with the labeled cell membrane. A short interval pre-bleaching (5 s) of the immobile fraction of fluorophores was followed by FCS data collection. The fluctuation of fluorescence intensity produced by the diffusion of labeled membrane HER2 molecules in this sub-femtoliter volume was measured, and an autocorrelation-function was generated:

$$G_{I\left(\tau \right)}=\frac{\langle I_{\left(t\right)}I_{\left(t+\tau \right)}\rangle }{{\langle I_{\left(t\right)}\rangle }^{2}},$$

where *t* indicates any time point during acquisition and *τ* indicates the various time delays for which correlation was computed. The operator 〈〉 indicates averaging over the whole range of data. In the case of cross-correlation analysis (FCCS), HER2 molecules in the membrane were labeled with a 1:1 mixture of 76.5 Fabs conjugated with either Alexa-Fluor 488 or Alexa-Fluor 647. Confocal images demonstrate strong co-localization of the two spectrally distinct labels. Collection of intensity fluctuation time traces of both channels was followed by the generation of autocorrelation and cross-correlation functions. The cross-correlation function

$$G_{Ix\left(\tau \right)}=\frac{\langle I_{gr\left(t\right)}I_{r\left(t+\tau \right)}\rangle }{\langle I_{gr\left(t\right)}\rangle \langle I_{r\left(t\right)}\rangle }$$

is derived along principles and variables matching those in the autocorrelation function, except for the *I* intensity in one spectral channel (*gr* for green emission in our case), which is correlated with the signal in the other channel (*r* for red emission in our case) at various *τ* delays.

The FCS data collection time-period of one cell was 10 s repeated 10 times before moving to another cell in the same well. Owing to the time needed for finding the cell and the z position in the cell, one cell in every 2–3 min could be assessed using this method. The characteristic parameters of diffusion were calculated for the first 5 min interval post-stimulus. EGF stimulation (100 nM final concentration) was introduced after obtaining a few control traces (in ~15 min). Several rounds of starvation-labeling-measurement cycles were used to accumulate the data of 8–30 independent cells per treatment.

### 4.4. Analysis of FCS/FCCS Data

All fluctuation data were analyzed using Zeiss Aim 4.0 Sp2 and Zen Black 2.1 software. The following components were included for fitting the autocorrelation (and cross correlation) functions:

- The fast two-dimensional membrane component is attributed to labeled monomers or smaller/more mobile oligomers in the membrane.
- The slower but still two-dimensional membrane component is attributed to labeled higher oligomers/aggregates.
- The model also contains a <10 ms time constant for the three-dimensional diffusion of non-bound Fab fragments.

Overall, the model is described by

$$G_{tot\left(\tau \right)}=1+G_{triplet\left(\tau \right)}G_{D\left(3comp\right)\left(\tau \right)},$$

where

$$G_{triplet\left(\tau \right)}=\frac{\left(1-T+Te^{-\tau /\tau_{tr}}\right)}{1-T}$$

is the triplet component with an *τ_tr_* triplet lifetime, and molecular diffusion is characterized by

$$G_{D\left(3comp\right)\left(\tau \right)}=G_{free3D\left(\tau \right)}+G_{anomal2D\left(\tau \right)}=\frac{\phi_{1}}{\left(1+\frac{\tau }{\tau_{d1}}\right)\left(1+\frac{\tau }{\tau_{d1}}\frac{1}{S^{2}}\right)}+\sum_{i=2}^{3}\frac{\phi_{i}}{\left(1+{\left(\frac{\tau }{\tau_{di}}\right)}^{\alpha_{i}}\right)},$$

where $\phi_{i}$ is the fractional intensity of the *i*th component, $\tau_{di}$ is the diffusional correlation time of the *i*th component, S is a structural parameter relating the *z* axis of the observation volume to the *x*/*y* axes (~7 in our case), and $\alpha_{i}$ is an anomaly parameter for describing anomalous (hindered or facilitated) diffusion. The average number of diffusing species in the observation volume was calculated as 1/*G*_*anomal*2*D*(0)_. For evaluating cross-correlation, the triplet and free antibody components are omitted, as these are not expected to cross-correlate.

### 4.5. Generation of the Photon-Count Histogram (PCH)

The average photon count $\langle k\rangle$, i.e., brightness of the fluorescence species (where a monomer or a large aggregate equally counts as a single species) was calculated as $\langle k\rangle =\varepsilon \frac{V_{PSF}}{V_{0}}$, where ε, the molecular brightness, is defined as $\varepsilon =I_{0}^{N}\beta \eta_{w}T$.

Here, $V_{PSF}$ is the illumination volume, $V_{0}$ is the sample volume, T is the integration time increment, $\eta_{w}$ is the detection efficiency, $I_{0}$ is the maximum excitation when the fluorophore is at the center of the illuminated volume, and β is a constant incorporating excitation probability, quantum yield, and instrument bias.

### 4.6. Western Blot Analysis of EGFR and HER2 Tyrosine Phosphorylation

For immunoblotting, equal numbers of cells were seeded onto cell culture petri dishes (Eppendorf AG, Hamburg, Germany), left to adhere completely, and then serum-starved for 2 h. In the case of ligand stimulation, cells were incubated with EGF (100 nM) for 2, 5, or 10 min. In some of the experiments, pretreatment with pertuzumab, either whole antibody or Fab fragment (both at 50 μg/mL), were used for 20 min. The appropriate stimulation or pretreatment + stimulation protocol was followed by washing with ice-cold buffer and lysis immediately. Afterwards, cells were scraped up by a rubber policeman in lysis buffer (20 mM Tris, 1% NP-40, 137 mM NaCl, 2 mM EDTA, 10% glycerol, pH 8.0, and freshly added 2 mM Na-ortho-vanadate, 1 mM PMSF, dithiothreitol (DTT), protease inhibitor cocktail (cOmplete, Tablets Mini, Roche Holding AG, Basel, Switzerland)). An equal protein content of lysates was verified with Bradford reagent (B6916, Merck). Lysates were mixed and boiled with sample buffer and separated electrophoretically on 8% polyacrylamide gel in reducing SDS sample buffer using a Bi-Rad PowerPac HC (Bio-Rad Laboratories, Inc., Hercules, CA, USA) for approximately 1.5 h at 120 V. A wet blotting system (Bio-Rad) was used to transfer proteins onto PVDF-Immobilon P membranes (Merck Millipore, Burlington, MA, USA). Membranes were blocked with a 5% non-fat milk powder solution of 0.1% Tween20 containing TRIS based buffer (5% MLK-TTBS), for 1 h at room temperature. The primary antibodies (1–2 μg/mL in 1% MLK-TBS), which were applied for 1.5 h or overnight incubation, were anti-HER2 (cneuAB3/OP15, Sigma-Aldrich, St. Louis, MO, USA), anti-pHER2 (cerbB2 Ab18–PN2N, NeoMarker/Merck-Millipore), anti-EGFR (MAP clone F4, Thermo Fisher Scientific, Waltham, MA, USA), and anti-pEGFR (1H12, Cell Signaling Technology, Inc. Danvers, MA, USA). Samples were washed three times with 0.1% Tween-TBS followed by HRP-anti-mouse IgG conjugate as secondary antibody (1 μg/mL, in 1% MLK-TBS, 2 h at room temperature). Immunoreaction was detected using enhanced chemiluminescence (ECL) reagent (Supersignal West Pico, Thermo Fisher Scientific, Waltham, MA, USA) and captured with the chemiluminescence imaging system (Multilmage III, Alpha-Innotec, Kasendorf, Germany). Three independent experiments were performed.

### 4.7. Measurement of Cell Metabolic Activity

EZ4U (Biomedica Medizinprodukte GmbH, Vienna, Austria), an MTT-based assay, was applied to assess overall cell metabolic activity and its changes upon treatment by trastuzumab (Trazimera, Pfizer, New York, NY, USA) or pertuzumab. Cells were seeded onto 96-well microplates in indicator-free medium. The adhered cells were treated with either or none of the antibodies on day 1 at a final concentration range of 0.001 to 100 µg/mL in logarithmic steps. For all three regimes, controls as well as samples supplemented on days 1 and 3 with 100 nM EGF were run. On day 4, a Spectramax i3x multimode reader (TECAN SPARK, Männedorf, Switzerland) was used to read absorption at 450 and 620 nm 4 h after adding EZ4U, according to the manufacturer’s instructions. For each sample, 3 technical replicates were run in each of 3 or more independent experiments.

### 4.8. Statistical Evaluation of Data

FRET efficiency and fractional distribution related to oligomers were compared with ANOVA followed by Dunett’s multiple comparison test. Bartlett’s test was applied to verify equal variances. SigmaPlot 12 and GraphPad Prism 5.03 were used for analysis.

To analyze dose-effect curves, data were fitted with the Hill equation (inhibitory dose-response curve with variable slope model). For comparison of the efficacy of trastuzumab and pertuzumab, the data points in each experiment were normalized to the untreated controls (maximum growth rate), and the mean for each treatment and concentration was normalized to this value.

## Data Availability

Data is contained within the article and Supplementary Materials.

## Supplementary Materials

The following supporting information can be downloaded at https://www.mdpi.com/article/10.3390/ijms25115978/s1.

## Abbreviations

ADCC	Antibody dependent cellular cytotoxicity
EGF	Epidermal growth factor
EGFR	Epidermal growth factor receptor
FCS	Fluorescence correlation spectroscopy
FCCS	Fluorescence cross-correlation spectroscopy
FRET	Förster resonance energy transfer
HER2	Human epidermal growth factor receptor-2
PCH	Photon-count histogram
RiFRET	Ratiometric imaging FRET
TME	Tumor microenvironment
