# Supplementary material for: Disrupting EGFR–HER2 Transactivation by Pertuzumab in HER2-Positive Cancer: Quantitative Analysis Reveals EGFR Signal Input as Potential Predictor of Therapeutic Outcome
Source: Int J Mol Sci. 2024 May 29;25(11):5978. doi: 10.3390/ijms25115978 (PMC11173106; doi:10.3390/ijms25115978)
Supplement: Supplementary file 1 [file ijms-25-05978-s001.zip › ijms-2975515-supplementary.pdf]

Disrupting EGFR-HER2 Transactivation by Pertuzumab in HER2-positive Cancer: Quantitative Analysis Reveals EGFR Signal Input as Potential Predictor of Therapeutic Outcome

László Ujlaky-Nagy [1,2], János Szöllősi [1,2] and György Vereb [1,2,3,*]

Supplementary Materials:

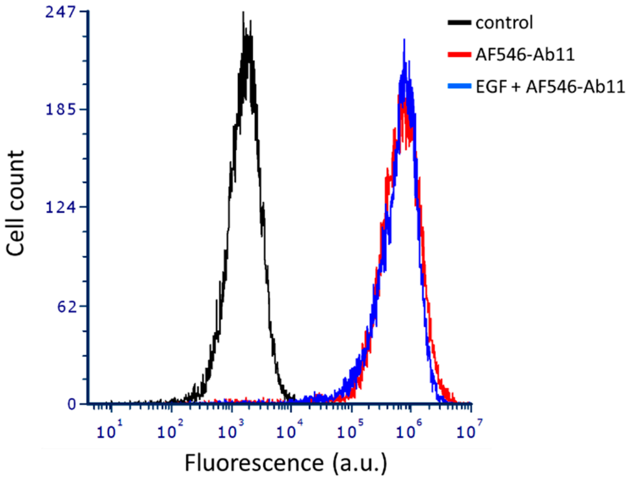

Figure S1: Non-competing nature of Ab11 with EGF binding

Equivalent binding of Ab11 in the absence or presence of EGF was confirmed to ensure that EGF stimulation does not cause unwanted labeling artifacts. A431 cells grown to 70% confluence over 48 h were harvested by standard TE detachment, washed twice in HBSS, and 200,000-cell aliquots were chilled in an icy waterbath in 50 µL HBSS. Labeling was done on ice with 20 µg/mL Alexa Fluor 546 conjugated Ab11 monoclonal antibody for 5 minutes only to avoid internalization of the receptor in the EGF-treated aliquot. This EGF + AF546-Ab11 sample was treated with 100 nm EGF for 1 minute before adding the antibody label. The control sample was mock-labeled with HBSS. Labeling was followed by washing once in ice-cold HBBS. Then formaldehyde was added at 1% (v/v) final concentration, and 20,000 cells from each sample were measured in a FACSAria III flow cytometer. FL2 histograms are displayed without gating.

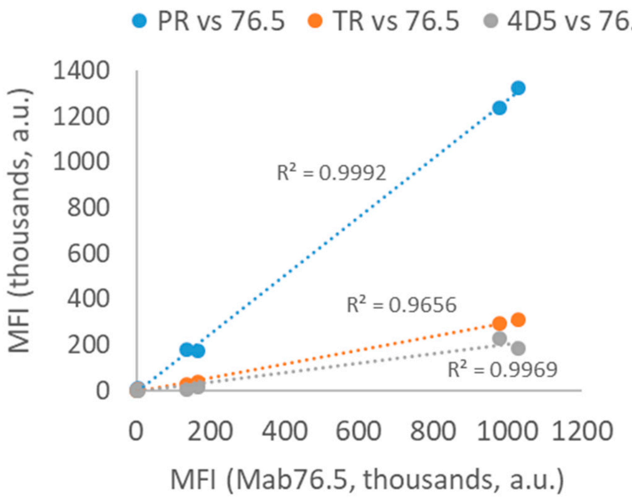

Figure S2: Mab 76.5 binding in comparison to trastuzumab and pertuzumab.
Binding of the Mab 76.5, prepared from hybridoma provided as a kind gift by Yosef Yarden (Weizmann Inst. of Science, Rehovot, IL) was compared to the binding of trastuzumab, its mouse parent antibody, 4D5, and pertuzumab on cell lines expressing various amounts of, or no HER2. All antibodies were directly conjugated with Alexa Fluor 647. Cells, grown to 70% confluence over 48 h were harvested by standard TE detachment, washed twice in HBSS, and 200,000-cell aliquots were chilled in an icy waterbath in 50 µL HBSS. Labeling was done on ice with 20 µg/mL Alexa Fluor 647 conjugated monoclonal antibodies for 10 minutes. Labeling was followed by washing once in ice-cold HBBS. Then formaldehyde was added at 1% (v/v) final concentration, and 20,000 cells from each sample were measured in a Novocyte RYB flow cytometer. FL3 histogram means (MFI) corrected with MFI for the corresponding mock-labeled cells are plotted for the various cell lines. The values for pertuzumab (PR), trastuzumab (TR) and 4D5, respectively, are plotted against the values for Mab 76.5 samples on the abscissa. The first set of points at (0;0), practically indistinguishable from each other, are derived from the negative control cell lines with no HER2 expression: the B lymphoblastoid JY, and the basal A type MDA-MB-468 and the basal B type MDA-MB-231 triple negative breast cancer lines. Low expressing cell lines were the HER2-transduced version of MDA-MB-468 (with 100,000 HER2/cell), and the JIMT-1 breast cancer line (130,000 HER2/cell). The high expressing group included the SK-BR-3 breast cancer (800,000 HER2/cell), and the N87 (850,000 HER2/cell) gastric cancer cell lines.
